# Supplementary material for: Mapping the evolution of fertility support policies in China: A content and instrumental analysis
Source: PLoS One. 2025 Oct 9;20(10):e0332137. doi: 10.1371/journal.pone.0332137 (PMC12510515; doi:10.1371/journal.pone.0332137)
Supplement: S1 Appendix — (ZIP) [file pone.0332137.s001.zip › S1 Appendix. 226 original policy documents/225-财政部、国家税务总局关于生育津贴和生育医疗费有关个人所得税政策的通知(FBM-CLI-4-103586).doc]

财政部、国家税务总局关于生育津贴和生育医疗费有关个人所得税政策的通知

**发布部门：** [财政部](https://www.pkulaw.com/law/chl?Aggs.IssueDepartment=60210) [国家税务总局](https://www.pkulaw.com/law/chl?Aggs.IssueDepartment=60401) [机构沿革](javascript:void(0))

**发文字号：**财税〔2008〕8号

**发布日期：**2008.03.07

**实施日期：**2008.03.07

**时效性：** [现行有效](https://www.pkulaw.com/law/chl?Aggs.TimelinessDic=01)

**效力级别：** [部门规范性文件](https://www.pkulaw.com/law/chl?Aggs.EffectivenessDic=XE0302)

**法规类别：** [计划生育统计财会](https://www.pkulaw.com/law/chl?Aggs.Category=09703) [个人所得税](https://www.pkulaw.com/law/chl?Aggs.Category=0320305)

财政部、国家税务总局关于生育津贴和生育医疗费有关个人所得税政策的通知
（财税[2008]8号　2008年3月7日）

各省、自治区、直辖市、计划单列市财政厅（局）、地方税务局，新疆生产建设兵团财务局：
　　根据《中华人民共和国个人所得税法》有关规定，经国务院批准，现就生育津贴和生育医疗费有关个人所得税政策通知如下：
　　**一、**生育妇女按照县级以上人民政府根据国家有关规定制定的生育保险办法，取得的生育津贴、生育医疗费或其他属于生育保险性质的津贴、补贴，免征个人所得税。

　　**二、**上述规定自发文之日起执行。

| 引用本篇的法规 案例 论文 |  |
| --- | --- |

| 部门规章 |  |
| --- | --- |

[财政部、税务总局关于继续有效的个人所得税优惠政策目录的公告](https://www.pkulaw.com/chl/6377043e2dc9407bbdfb.html)

[国家税务总局公告2015年第73号――关于发布《减免税政策代码目录》的公告](https://www.pkulaw.com/chl/cc7e445488b3f5a9bdfb.html)

[财政部注册会计师考试委员会关于发布《注册会计师全国统一考试大纲——专业阶段考试(2013年度)》和《注册会计师全国统一考试大纲——综合阶段考试(2013年度)》的通知](https://www.pkulaw.com/chl/7f70220f06b26052bdfb.html)

[审计署审计结果公告2012年第34号——全国社会保障资金审计结果](https://www.pkulaw.com/chl/06517194028b215bbdfb.html)

[财政部注册会计师考试委员会关于发布《注册会计师全国统一考试大纲——专业阶段考试(2012年度)》和《注册会计师全国统一考试大纲——综合阶段考试(2012年度)》的通知](https://www.pkulaw.com/chl/6834e99b62690279bdfb.html)

[财政部注册会计师考试委员会关于印发《注册会计师全国统一考试大纲－－专业阶段考试(2010年度)》的通知](https://www.pkulaw.com/chl/4ab8dc2264763530bdfb.html)

| 地方法规规章 |  |
| --- | --- |

[河北省财政厅关于公布规范性文件清理结果的通知(2021)](https://www.pkulaw.com/lar/024dcd2452ce0d499c8dc9d51cef41dbbdfb.html)

[安徽省财政厅、安徽省国家税务局、安徽省地方税务局关于发布《安徽省财税优惠事项清单》的公告](https://www.pkulaw.com/lar/1d1b42f9f90f03e5ed3a5dee9da3051fbdfb.html)

[江西省地方税务局关于发布有效、失效、废止的税收规范性文件目录的公告(2013)](https://www.pkulaw.com/lar/02212b849ff688bd144149f9cd7e835abdfb.html)

[江西省地方税务局关于发布有效、失效、废止的税收规范性文件目录的公告](https://www.pkulaw.com/lar/042245718fc83e1f75134589a7b7ee7abdfb.html)

[广州市地方税务局关于印发《个人所得税若干征税业务指引(2009年)》的通知](https://www.pkulaw.com/lar/25cc94e68adec35e6259f0444cc8836abdfb.html)

[上海市地方税务局关于转发《财政部、国家税务总局关于生育津贴和生育医疗费有关个人所得税政策的通知》的通知](https://www.pkulaw.com/lar/727c547773c9e0201102d8c037b82cd3bdfb.html)

| 案例与裁判文书 |  |
| --- | --- |

[广州王老吉大健康产业有限公司、安丽玲劳动争议二审民事判决书](https://www.pkulaw.com/pfnl/a6bdb3332ec0adc4414f78cfe6a22e8fb7b23f9810d3d744bdfb.html)

© 北大法宝：（[www.pkulaw.com](https://www.pkulaw.com/)) 专业提供法律信息、法学知识和法律软件领域各类解决方案。北大法宝为您提供丰富的参考资料，正式引用法规条文时请与标准文本核对。
欢迎查看所有[产品和服务](https://www.pkulaw.com/helps/2.html)。法宝快讯：[如何快速找到您需要的检索结果？](https://www.pkulaw.com/helps/70.html)[法宝](https://www.pkulaw.com/helps/69.html)V6有何新特色？

扫描二维码阅读原文

**原文链接：**<https://www.pkulaw.com/chl/1826fee2ed2ee421bdfb.html>
